# Physical Health, Media Use, and Mental Health in Children and Adolescents With ADHD During the COVID-19 Pandemic in Australia

**DOI:** 10.1177/1087054720978549

**Published:** 2020-12-17

**Authors:** Emma Sciberras, Pooja Patel, Mark A. Stokes, David Coghill, Christel M. Middeldorp, Mark A. Bellgrove, Stephen P. Becker, Daryl Efron, Argyris Stringaris, Stephen V. Faraone, Susannah T. Bellows, Jon Quach, Tobias Banaschewski, Jane McGillivray, Delyse Hutchinson, Tim J. Silk, Glenn Melvin, Amanda G. Wood, Anna Jackson, George Loram, Lidia Engel, Alicia Montgomery, Elizabeth Westrupp

**Affiliations:** 1 Deakin University, Geelong, VIC, Australia; 2 Murdoch Children’s Research Institute, Parkville, VIC, Australia; 3 University of Melbourne, Parkville, VIC, Australia; 4 The Royal Children’s Hospital, Parkville, VIC, Australia; 5 Child Health Research Centre, University of Queensland, Brisbane, QLD, Australia; 6 Child and Youth Mental Health Service, Children’s Health Queensland Hospital and Health Service, Brisbane, QLD, Australia; 7 Turner Institute for Brain and Mental Health, Monash University, Clayton, VIC, Australia; 8 Cincinnati Children’s Hospital Medical Center, OH, USA; 9 University of Cincinnati College of Medicine, OH, USA; 10 National Institute of Mental Health, Rockville, MD, USA; 11 State University of New York Upstate Medical University, Syracuse, USA; 12 Central Institute of Mental Health, Heidelberg University, Germany; 13 University of New South Wales, Kensington, NSW, Australia; 14 School of Life Sciences, Aston University, Birmingham, UK; 15 Judith Lumley Centre, La Trobe University, Bundoora, VIC, Australia

**Keywords:** ADHD, COVID-19, psychological well-being

## Abstract

**Objective:**

To examine the impact of COVID-19 restrictions among children with attention-deficit/hyperactivity disorder (ADHD).

**Methods:**

Parents of 213 Australian children (5–17 years) with ADHD completed a survey in May 2020 when COVID-19 restrictions were in place (i.e., requiring citizens to stay at home except for essential reasons).

**Results:**

Compared to pre-pandemic, children had less exercise (Odds Ratio (OR) = 0.4; 95% CI 0.3–0.6), less outdoor time (OR = 0.4; 95% 0.3–0.6), and less enjoyment in activities (OR = 6.5; 95% CI 4.0–10.4), while television (OR = 4.0; 95% CI 2.5–6.5), social media (OR = 2.4; 95% CI 1.3–4.5), gaming (OR = 2.0; 95% CI 1.3–3.0), sad/depressed mood (OR = 1.8; 95% CI 1.2–2.8), and loneliness (OR = 3.6; 95% CI 2.3–5.5) were increased. Child stress about COVID-19 restrictions was associated with poorer functioning across most domains. Most parents (64%) reported positive changes for their child including more family time.

**Conclusions:**

COVID-19 restrictions were associated with both negative and positive impacts among children with ADHD.

The COVID-19 pandemic is a global health emergency that has major potential ramifications for health and well-being (Holmes et al., 2020). Emerging research points to impacts on the health and well-being of young people, but little research has examined impacts among potentially higher-risk groups, such children and adolescents (referred to as children thereafter) with Attention-Deficit/Hyperactivity Disorder (ADHD). Understanding the impacts for specific clinical subgroups will help ensure that supports are tailored to meet their specific needs.

It is plausible that COVID-19 may be particularly challenging for children with ADHD (Cortese et al., 2020; Cortina et al., 2020; McGrath, 2020; Waite et al., 2020). Most children with ADHD have one or more comorbid conditions, such as anxiety and mood disorders, disruptive behavior disorders (e.g., Oppositional Defiant Disorder [ODD], Conduct Disorder [CD]), and Autism Spectrum Disorder (ASD)) (Faraone et al., 2015). The changes in routine, structure, and social contact associated with COVID-19 restrictions, as well as increasing uncertainty, may exacerbate ADHD symptoms and associated problems (Cortina et al., 2020). Furthermore, more time inside and fewer opportunities for exercise may contribute to a worsening of sleep problems (Becker & Gregory, 2020) and increased media use in children with ADHD; aspects of life that are that are commonly challenging for families of children with ADHD to regulate (Cortese et al., 2013; Thoma et al., 2020). Positives may also be experienced as a result of the COVID-19 restrictions. Spending more time at home, particularly attending school from home, may remove some school-related stressors that children with ADHD commonly experience, such as frustration related to underachievement and social difficulties (Becker et al., 2017; Harpin et al., 2016; Loe & Feldman, 2007).

It is unclear how the pandemic may be impacting the healthcare of children with ADHD. In countries around the world, face-to-face services have reduced, with the provision of services via telehealth (i.e., consultations via telephone or videoconferencing) being commonplace (Badawy & Radovic, 2020; Cortese et al., 2020). There is a lack of clarity as to whether government restrictions in Australia have affected the quality of care and efficacy of treatments provided by practitioners. Although best practice guidance suggests children with ADHD should continue their prescribed medication and dosage during the pandemic to aid children in following the physical distancing recommendations (Cortese et al., 2020), it is unclear whether these recommendations are being followed. It is also unclear what additional support parents may wish to receive during this period.

One study from Shanghai examined the experience of children with ADHD during COVID-19 restrictions (*N* = 241 parents of children with ADHD; Zhang et al., 2020). The study found evidence of children’s ADHD-related behaviors worsening during the pandemic compared to their usual functioning. Notably, 54% of parents reported their child’s ability to focus had worsened, 67% had increased anger levels, and 56% had worse daily routines. Conversely, 30% to 40% of parents reported that their child’s sleep and eating had improved during the pandemic. Similarly, a study of children with ADHD in lockdown in France found that 34% of children were reported to have worse wellbeing, while 31% were reported to be functioning better (Bobo et al., 2020). However, these studies did not systematically report on life changes due to COVID-19, nor changes in broader behaviors, such as physical activity, media use, anxiety, and/or loneliness. Furthermore, disruptions to healthcare use/availability and parent preferences for support during this period were not examined. In addition, factors associated with worse functioning during this time such as COVID-19 related stress and worries (e.g., stress associated with adhering to restrictions, worry about contracting COVID-19), relative to other clinical factors, such as comorbidities and ADHD medication use were not examined.

The present study aimed to understand the impact of COVID-19 on Australian families of children with ADHD. In Australia, the first COVID-19 case was identified in January 2020 (Department of Health, 2020), with the number of new cases rising in March and then slowing to a relatively low number of daily reported cases in mid-April (Australian Government Department of Health, 2020). The current study collected data over a 4-week period in May 2020. As at May 1, 2020, rates of COVID-19 infections were lower in Australia than many other high-income countries, with a total of 6,762 confirmed cases and 92 deaths reported (incidence rate of 26.52 cases and 0.36 deaths per 100,000 people; World Health Organization, 2020). By the beginning of May 2020, social distancing measures were in effect with residents required to work from home if possible and expected to stay at home except for essential reasons such as shopping for food and accessing medical care. Most Australian states required children to learn from home, although children of essential workers were permitted to physically attend school. Towards the end of May, a staggered relaxing of social distancing including physical school attendance was implemented in most states, with all children expected to physically return to school by early June.

Specifically, the current study aimed to examine the following in a sample of children with ADHD:

1. Life changes due to the COVID-19 restrictions including impacts on parent work, finances, and social relationships;
2. Differences in child physical health, media use and mental health, before and during the pandemic;
3. Whether children’s COVID-19 stress and worries were associated with negative changes in physical health, media use, and mental health, before and during the pandemic, relative to clinical factors, such as comorbidities and medication use;
4. Changes or barriers to healthcare during COVID-19 restrictions; and
5. Parent interest in online interventions during COVID-19 restrictions.

## Method

### Study Design and Eligibility

This study used baseline data from the ADHD COVID-19 survey, a longitudinal study surveying parents of children with ADHD during the COVID-19 pandemic. Two hundred and twenty-one parents consented to participate in the study. Eligible parents were aged 18 years or above with a child aged between 5 and 17 years who had been diagnosed with or treated for ADHD and were living in Australia. To be included in analyses, participants needed to have data available on at least one variable of interest (*n* = 213). The N included in all analyses is footnoted in each table. The study had ethical approval from Deakin University (HEAG-H 60_2020).

### Procedure

This study was advertised through ADHD organizations and support groups in Australia. Organizations emailed the study advertisements to families and posted the advertisement on their social media sites. Interested parents clicked a link to read the participant information and provide their consent to complete an online survey in REDCap (Research Electronic Data Capture) (Harris et al., 2009; Harris et al., 2019).

### Measures

#### Child physical health, media use, and mental health

We assessed child physical health, media use, and mental health by parent report using the CoRonavIruS Health Impact Survey (CRISIS) (Nikoladis et al., 2020). Parents provided two ratings in separate sections of the online survey; one rating based on functioning 3 months prior to the pandemic (16 items) and one rating based on functioning in the last 2 weeks (16 items). Pre and current areas of functioning assessed were physical health (sleep, physical activity), media use (TV, social media, and gaming), and mental health (e.g., negative thoughts, loneliness, and depressed mood; see Tables 2 and 3 for a list of all domains assessed).

#### Life changes due to COVID-19

**Figure 1.**
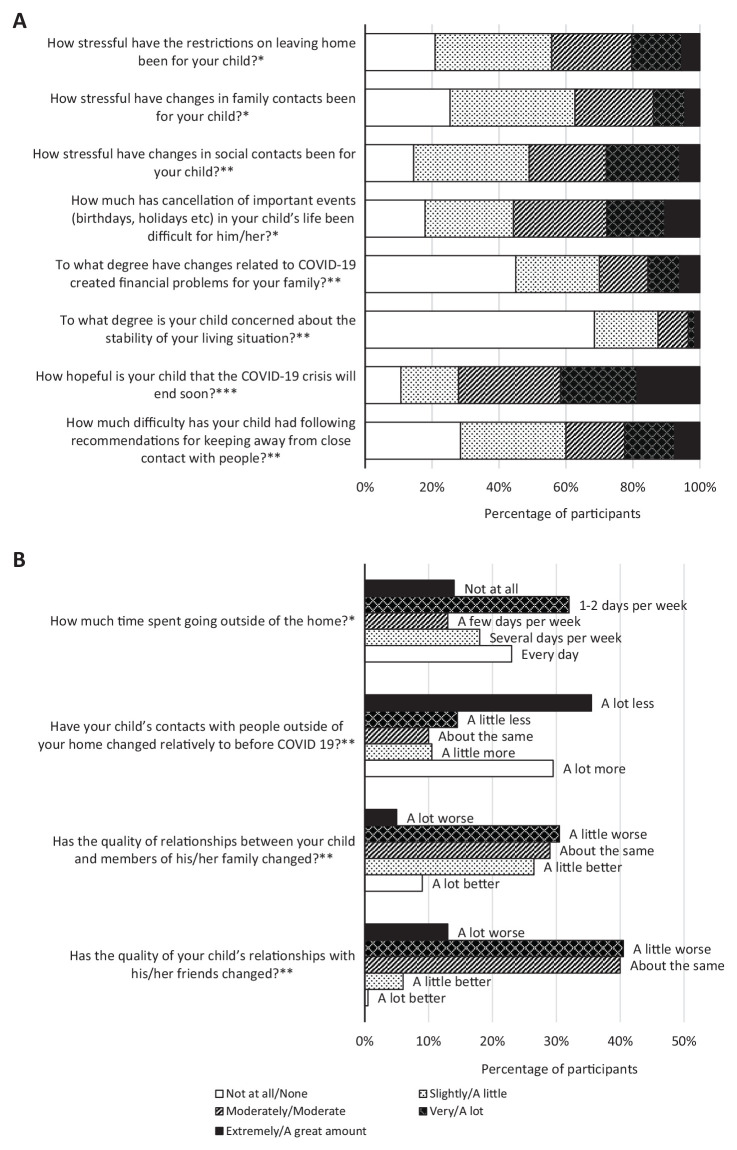
Life changes due to COVID-19 related restrictions. *Note*. *N = 201. **N = 200. ***N = 197.

Twelve items from the CRISIS (Nikoladis et al., 2020) were used to assess life-changes due to the COVID-19 restrictions (e.g., stress associated with restrictions; see Figure 1 for a full list of the questions). In addition, parents also responded to an open-ended question about the positive impacts of the COVID-19 restrictions. We used questions originally developed for the COVID-19 Pandemic Adjustment Survey (CPAS) (Westrupp et al., 2020) to assess changes to parent work. Parents were asked “Has your work situation changed since the COVID-19 pandemic?” and selected all applicable changes including loss of job, changed work hours, new roles/responsibilities, and working from home. Parents reported on the same questions for their partner, if applicable.

#### Changes or barriers to healthcare

Study designed questions assessed changes or barriers to healthcare for children in the past month including difficulty accessing medication, whether their child had stopped taking medication, changes in their child’s medication dose, and whether the child had started a new prescribed medication. If parents reported “yes” to any of these changes, they were asked to describe the change (free text field). Parents reported the number of visits to health professionals, whether they had difficulties accessing healthcare services, and rated the quality of telehealth services, if applicable.

#### Parent interest in online interventions

Adapted from CPAS (Westrupp et al., 2020), parents indicated how likely (on a five point scale ranging from “not at all” to “extremely likely”) they would be to use an online or smartphone intervention for: (1) mental health support for their child; (2) parenting support; (3) education support for their child; and (4) sleep support for their child. Parents then indicated how likely they were to use a self-guided or therapist-assisted internet or smartphone app-based program for support.

#### Child COVID-19 stress and worry

We used 4 items from the CRISIS (Nikoladis et al., 2020) to create a composite measure of child stress associated with COVID-19 restrictions, comprised of the following questions: (1) how stressful the restrictions on leaving home have been for the child; (2) how stressful changes in family contacts have been for the child; (3) how stressful changes in friend contacts have been for the child; and (4) difficulty experienced with cancellation of events. We used two items from the CRISIS ((1) child worry about becoming infected; and (2) child worry about friends or family being infected) to generate a composite of child worry about COVID-19. All items were rated on a five-point scale and for each domain an average score was created with higher scores indicating more stress (COVID-19 stress α = 0.81; COVID-19 worry α = 0.84). Model fit statistics from a confirmatory factor analysis using maximum likelihood estimation supported the goodness of fit of our theorized model (Hu & Bentler, 1999): *χ*^2^ = 12.14, *p* = .15; Comparative Fit Index (CFI) = 0.99; Tucker–Lewis Index (TLI) = 0.98; Root Mean Square Error of Approximation (RMSEA) = 0.05; see Supplemental Figure 1. The measurement model was assessed and no Heywood cases were detected.

#### Demographics

Parents reported on their place of birth, language spoken at home, Aboriginal or Torres Strait Islander status, age, education level, employment, partner status, number of children in the home, and whether they were the child’s biological parent. If the parent had a partner, they reported on their partner’s education level and employment. Parents also reported on their child’s age, sex, comorbid conditions, and medication use.

#### Statistical analyses

Descriptive statistics were used to describe the characteristics of the sample, as well as the life changes associated with COVID-19 (aim 1). Differences in physical health, media use, and mental health three months prior to COVID-19 compared to present functioning were analyzed using proportion tests (aim 2). Odds ratios and 95% CIs are reported. For this aim, domains of functioning were dichotomized to reflect potentially problematic functioning. For example, collapsing categories reflecting a symptom being present at least moderately, compared to the symptom being present not at all or only slightly. Dichotomization of other variables followed best practice recommendations, for example, less than 8 hours of sleep per night compared with 8 hours or more sleep per night. Given the number of statistical comparisons conducted for aim 2, we undertook a false discovery rate (FDR) correction (Benjamini & Hochberg, 1995). For aim 3, we compared parent ratings 3 months prior to the pandemic to current ratings of functioning and coded children as 0 = No change/positive change in functioning or 1 = Negative change in functioning, across the 16 domains. We then examined the association between child COVID-19 stress and worries (entered simultaneously in the same model) and change in functioning across the 16 domains using adjusted logistic regression analyses accounting for child age, child sex, ADHD medication use, externalizing disorder diagnosis by parent report, internalizing disorder diagnosis by parent report, neighborhood socio-economic status, and financial insecurity (single item from CRISIS ( Nikoladis et al., 2020): To what degree have changes related to COVID-19 created financial problems for your family, rated on a five-point scale from 1 “Not at all” to 5 “Extreme”). Again, given the number of statistical comparisons for this aim, we undertook an FDR correction. We also conducted supplementary analyses, repeating all analyses using adjusted linear regression treating change in functioning dimensionally. Descriptive statistics were used to summarize health service impacts (aim 4) and parent interest in online interventions (aim 5).

## Results

### Sample Characteristics

The mean age of the children was 11 years (*SD* = 3.09) and most were male (76.4%) (Table 1). Comorbidities were common with over half reported to have an anxiety disorder (59.5%) and one third a learning disorder (28.3%). Most children were taking medication to assist with learning, behavior, emotions or sleep, most commonly a medication for ADHD (84.6%). About 1 in 5 were taking an antidepressant medication (19.2%), while nearly half were taking melatonin (43.9%). The majority of respondents were female (97.7%) and had completed high school (87.8%) and university education (61.0%). Most primary caregivers reported having a partner (76.5%) and being employed prior to COVID-19 (77.8%). Most partners were reported to have completed high school (71.2%), with under half having completed university (39.9%).

**Table 1.** Sample Characteristics.

Characteristics	*N*	%
Primary caregiver characteristics^a^		
Born in Australia	171	82.6
Aboriginal or Torres Strait Islander	5	2.4
Primary caregiver female	208	97.7
Primary caregiver age, *M* (*SD*), range	42.57 (5.6), 27–56	
Completed high school	187	87.8
Completed university	130	61.0
Employed prior to COVID-19	165	77.8
Has a partner	163	76.5
Child’s biological parent	210	98.6
Partner characteristics^b^		
Partner completed high school	116	71.2
Partner completed university	65	39.9
Partner employed prior to COVID-19	158	96.9
Family characteristics^c^		
English spoken at home	206	96.7
Number of children in household, *M* (*SD*), range	2.15 (0.8), 1–5	
Child characteristics^d^		
Child age	10.59 (3.1), 5–17	
Male	162	76.4
Child comorbid conditions^e^		
Autism Spectrum Disorder	30	17.5
Learning Disorder	49	28.3
Depression	19	11.2
Anxiety	103	59.5
ODD	32	18.6
CD	2	1.2
OCD	12	7.0
Tourette’s Syndrome/Tics	9	5.3
Speech/Language Disorder	32	18.8
Child medication use^f^		
Any medication for learning, behavior, emotions, or sleep	152	88.4
ADHD medication^g^	148	84.6
Antidepressant medication^h^	32	19.2
Risperidone	10	5.9
Clonidine	17	10.0
Melatonin	75	43.9

*Note*. ^a^*n* = 207–213.

b *n* = 163.

c *n* = 213.

d *n* = 212–213.

e *n* = 169–173.

f *n* = 175.

g Stimulant medications, Atomoxetine, Guanfacine.

h Sertraline, Fluoxetine.

### Life Changes Due to COVID-19 Restrictions

None of the children nor their household family members were reported to have been diagnosed with COVID-19. Three children had a non-household family member diagnosed. About 11% had a family member put into self-quarantine. During the previous 2 weeks, parents reported 29% of children (62 out of 213) were moderately/very/extremely worried about being infected with COVID-19 themselves, and 27% (57 out of 213) were moderately/very/extremely worried about family or friends being infected.

Forty-four percent of parents reported that the restriction on leaving home had been moderately/very/extremely stressful for their child (see Figure 1). Thirty-six percent and 54% reported the quality of family and social relationships, respectively, were a little/a lot worse. About half of parents reported that their work situation had changed since the pandemic (Table 2). Sixty-four percent (*n* = 136) of parents reported that COVID-19 restrictions had also led to positive changes in their child’s life, including more time with family, being able to learn at home without distractions, parents being able to help with learning, being less busy, more relaxed, having more home time, and less pressure/stress related to going to school.

**Table 2.** Changes in Work Situation for Parents^a^ and Partners^b^ in Paid Work Prior to the COVID-19 Pandemic.

	Parent *N* (%)	Partner *N* (%)
Work situation changed	77 (47.0)	67 (42.4)
Lost job	12 (7.3)	5 (3.2)
Work hours reduced	25 (15.2)	21 (13.3)
Work hours increased	11 (6.7)	4 (2.5)
New roles/responsibilities	22 (13.4)	11 (7.0)
Working from home	41 (25.0)	39 (24.7)

*Note*. ^a^165 parents were in paid employment prior to the COVID-19 pandemic. *N* = 164.

b 158 parents reported their partner was in paid employment prior to the COVID-19 pandemic. *N* = 158.

### Differences in Child Physical Health, Mental Health, and Media Use Before and During the Pandemic

Parents reported several areas of child functioning that had declined during the pandemic compared to three months prior, including less regular exercise (OR = 0.4, 95% CI 0.3, 0.6), less outdoor time (OR = 0.4, 95% CI 0.3, 0.6), increased TV time (OR = 4.0, 95% CI 2.5, 6.5), increased social media use (OR = 2.4, 95% CI 1.3, 4.5), increased gaming (OR = 2.0, 95% CI 1.3, 3.0), increased sad/depressed/unhappy mood (OR = 1.8, 95% CI 1.2, 2.8), reduced enjoyment in usual activities (OR = 6.5, 95% CI 4.0, 10.4), and increased loneliness (OR = 3.6, 95% CI 2.3, 5.5) (see Table 3, Supplemental Table 1).

**Table 3.** Differences in Current Child Physical Health, Media Use, and Mental Health Compared to 3 months prior to COVID-19 (*n* = 172–179).^a^

	Pre-COVID-19 *N* (%)	Current *N* (%)	% difference (95% CI)	OR (95% CI)	*p*-value
Physical health					
<8 hours of sleep per day	71 (39.7)	70 (39.1)	0.6 (–9.6, 10.7)	1.0 (0.7, 1.6)	.91
<5 days of exercise per week	91 (50.8)	130 (72.6)	−21.8 (–31.6, –12.0)	0.4 (0.3, 0.6)	<.001^b^
<5 days outdoor per week	53 (29.6)	92 (51.4)	−21.8 (–31.7, –11.9)	0.4 (0.3, 0.6)	<.001^b^
Media use					
More than 1 hour of TV per day	143 (80.8)	161 (91.0)	−10.2 (–17.3, –3.0)	4.0 (2.5, 6.5)	.006^b^
More than 1 hour of social media per day	33 (18.8)	52 (29.6)	−10.8 (–19.7, –1.9)	2.4 (1.3, 4.5)	.02^b^
More than 1 hour of gaming per day	75 (42.4)	105 (59.3)	−16.9 (–27.2, –6.7)	2.0 (1.3, 3.0)	.001^b^
Mental health					
Moderately, very, or extremely worried	81 (46.6)	87 (50.0)	−3.4 (–13.9, 7.0)	1.2 (0.8, 1.8)	.52
Moderately, very sad/depressed/unhappy	49 (28.2)	72 (41.4)	−13.2 (–23.1, –3.3)	1.8 (1.2, 2.8)	.01^b^
Not at all/slightly enjoyed activities	35 (20.2)	107 (61.9)	−41.6 (–51.0, –32.2)	6.5 (4.0, 10.4)	<.001^b^
Moderately, very anxious/nervous	94 (54.7)	95 (55.2)	−0.6 (–11.1, 9.9)	1.0 (0.7, 1.6)	.91
Moderately, very, extremely fidgety	131 (75.7)	136 (78.6)	−2.9 (–11.7, 6.0)	1.2 (0.7, 1.9)	.52
Moderately, very, extremely fatigued	85 (49.1)	103 (59.5)	−10.4 (–20.8, 0.0)	1.5 (1.0, 2.3)	.05
Moderately or very unfocused/distracted	104 (59.8)	115 (66.1)	−6.3 (–16.4, 3.8)	1.3 (0.8, 2.0)	.22
Moderately, very, extremely irritable	106 (60.9)	120 (69.0)	−8.0 (–18.0, 1.9)	1.4 (0.9, 2.2)	.12
Moderately, very, extremely lonely	46 (26.4)	97 (55.8)	−29.3 (–39.2, –19.4)	3.6 (2.3, 5.5)	<.001^b^
Often, a lot of the time expressing negative thoughts	40 (23.0)	48(27.6)	−4.6 (–13.7, 4.5)	1.3 (0.8, 2.0)	.32

*Note*. ^a^Participants were included in analyses if they provided a pre-COVID-19 and current rating of functioning.

b *p*-values below FDR threshold of *p* = .025.

### Association Between Child COVID-19 Worries and COVID-19 Stress and Changes in Physical Health, Mental Health, and Media Use Before and During the Pandemic

In adjusted analyses, there was little evidence of a unique association between COVID-19 worries and COVID-19 stress and negative changes in sleep, physical activity, and outdoor time (Table 4), with no associations surviving FDR correction. No clinical factors were associated with changes in sleep, physical activity or outdoor time.

**Table 4.** Association Between COVID-19 Stress and Worries and Changes (Current Functioning Compared to 3 months Prior to Pandemic) In Physical Health, Media Use, and Mental Health.

	No change/positive change	Negative change	Adjusted OR for COVID-19 worries^a,b,c^		Adjusted OR for COVID-19 stress^a,d^	
	*N* (%)	*N* (%)	OR (95% CI)	*p*	OR (95% CI)	*p*-value
Physical health						
Sleep duration	144 (80.45)	35 (19.6)	1.6 (1.1, 2.5)	.02	1.5 (0.9, 2.4)	.10
Physical activity	90 (50.3)	89 (49.7)	0.8 (0.6, 1.2)	.32	1.5 (1.0, 2.2)	.04
Outdoor time	98 (54.8)	81 (45.3)	1.1 (0.8, 1.5)	.67	1.5 (1.0, 2.1)	.05
Media use						
Television	104 (58.8)	73 (41.2)	1.2 (0.8, 1.7)	.40	1.4 (0.9, 2.0)	.09
Social media	138 (78.4)	38 (21.6)	0.8 (0.5, 1.2)	.27	2.1 (1.3, 3.3)	.003^e^
Gaming	119 (67.2)	58 (32.8)	1.6 (1.1, 2.3)	.02	0.9 (0.6, 1.3)	.56
Mental health						
General worry	122 (70.1)	52 (29.9)	1.3 (0.9, 1.9)	.16	2.0 (1.3, 3.1)	.001^e^
Sad/depressed	109 (62.6)	65 (37.4)	0.9 (0.6, 1.3)	.57	3.0 (1.9, 4.8)	<.001^e^
Enjoying activities	63 (36.4)	110 (63.6)	0.9 (0.6, 1.4)	.75	3.1 (1.9, 5.0)	<.001^e^
Anxious/nervous	123 (71.5)	49 (28.5)	0.8 (0.5, 1.2)	.29	2.5 (1.6, 4.0)	<.001^e^
Fidgety	114 (65.9)	59 (34.1)	1.1 (0.7, 1.6)	.68	2.6 (1.7, 4.1)	<.001^e^
Fatigue	108 (62.4)	65 (37.6)	0.7 (0.5, 1.1)	.09	2.3 (1.5, 3.6)	<.001^e^
Distractibility	113 (64.9)	61 (35.1)	0.7 (0.5, 1,1)	.11	2.1 (1.4, 3.1)	<.001^e^
Irritable	99 (56.9)	75 (43.1)	0.8 (0.5, 1.2)	.27	2.8 (1.8, 4.4)	<.001^e^
Lonely	86 (49.4)	88 (50.6)	0.9 (0.6, 1.3)	.60	3.7 (2.2, 6.1)	<.001^e^
Negative thoughts	131 (75.3)	43 (24.7)	0.6 (0.4, 1.0)	.04	4.0 (2.3, 6.9)	<.001^e^

*Note*. ^a^*N* = 165–168.

b No associations surviving FDR correction.

c Adjusted for COVID-19 stress, child age, child sex, ADHD medication use, externalizing disorder, internalizing disorder, neighborhood socio-economic status, and financial insecurity.

d Adjusted for COVID-19 worries, child age, child sex, ADHD medication use, externalizing disorder, internalizing disorder, neighborhood socio-economic status, and financial insecurity.

e *p*-values below FDR threshold of *p* = .034.

In adjusted analyses (Table 4), child COVID-19 stress was significantly associated with increased social media use (AOR = 2.1; 95% CI 1.3, 3.3; *p* = .003). Although there was some evidence of an association between COVID-19 worries and increased gaming (AOR = 1.6; 95% CI 1.1, 2.3; *p* = .02), this finding did not survive FDR correction. In adjusted models, boys had higher odds of increased gaming (AOR = 2.5; 95% CI 1.0, 6.0; *p* = .04) and decreased social media use (AOR = 0.4; 95% CI 0.2, 1.0; *p* = .05) relative to girls. Older children also had higher odds of increased social media use (AOR = 1.2; 95% CI 1.0, 1.3; *p* = .05). No other clinical factors were independently associated with changes in media use.

In adjusted models, there was little evidence of an association between COVID-19 worries and negative changes in general mental health. In contrast, child COVID-19 stress was associated with increased general worry (AOR = 2.0; 95% CI 1.3, 3.1; *p* = .001), sadness (AOR = 3.0; 95% CI 1.9, 4.8; *p* < .001), anxiety/nervousness (AOR = 2.5; 95% CI 1.6, 4.0; *p* < .001), fidgety behavior (AOR = 2.6; 95% CI 1.7, 4.1; *p* < .001), fatigue (AOR = 2.3; 95% CI 1.5, 3.6; *p* < .001), distractibility (AOR = 2.1; 95% CI 1.4, 3.1; *p* < .001), irritability (AOR = 2.8; 95% CI 1.8, 4.4; *p* < .001), loneliness (AOR = 3.7; 95% CI 2.2, 6.1; *p* < .001), and negative thoughts (AOR = 4.0; 95% CI 2.3, 6.9; *p* < .001); see Table 4. Furthermore, COVID-19 stress was associated with decreased enjoyment in activities (AOR = 3.1; 95% CI 1.9, 5.0, *p* < .001). Children with an internalizing disorder had increased distractibility during the pandemic (AOR = 2.5; 95% CI 1.1, 5.5; *p* = .03), however, no other clinical factors were independently associated with negative changes across the mental health areas assessed.

All analyses were repeated treating change in functioning dimensionally (see Supplemental Table 2). Results were consistent with the exception that COVID-19 stress was associated with decreased physical activity (*p* = .001) and decreased outdoor time (*p* = .01) in adjusted models.

### Changes or Barriers to Healthcare

Of the 152 parents who reported their child was taking medication to assist with learning, behavioral, emotional or sleep difficulties, 11% (*n* = 16) reported difficulties accessing medication over the past month, with the most common reasons being medication out of stock (*n* *=* 8) and difficulty obtaining prescriptions (*n* = 4). Seventeen percent (*n* = 26) of those children taking medication had a dosage change over the past month (majority had increased dosage), and 11% (*n* = 16) started a new prescribed medication. Sixteen percent of parents (*n* = 28) reported that their child had stopped taking a medication over the past month, with most of the stopped medications being ADHD medications (*n* = 24). Reasons for stopping an ADHD medication included taking a break during school holidays (*n* = 5), not requiring medication due to school closure/remote learning (*n* = 6), and stopping one ADHD medication to start another (*n* = 3). Forteen out of the 24 children (58%) who had stopped taking an ADHD medication continued to take other medications.

Overall, 13% reported difficulty accessing healthcare services for their child in the past month. Table 5 outlines the healthcare use reported by parents in the last month. About one third of children were reported to have seen a pediatrician (33.9%) and/or a psychologist (32.1%) in the past month. Of the 107 families that used telehealth services, 42% rated telehealth as being the same quality as face-to-face appointments and 48% rated it as poorer quality.

**Table 5.** Healthcare Appointments Related to Difficulties With Learning, Behavior, or Emotions in the Past Month.^a,b^

	*N* (%) children who saw this professional	Mean number of visits (range)
G.P. or Family doctor	35 (20.4)	1.4 (1–4)
Pediatrician	58 (33.9)	1.1 (1–3)
Psychiatrist	17 (10.4)	1.5 (1–5)
Psychologist	54 (32.1)	2.0 (1–5)
Occupational therapist	19 (11.4)	2.3 (1–4)
Speech pathologist	23 (13.7)	2.5 (1–4)

*Note*. ^a^In-person or telehealth appointments.

b *N* = 164–172.

### Parent Interest in Online or Smartphone Interventions

Sixty-nine percent of parents (*n* = 100) reported being moderately/very/extremely likely to use an online or smartphone intervention for child mental health support, 70% (*n* = 100) were at least moderately likely to use a parenting support intervention, 69% (*n* = 100) were likely to use an intervention for child education support, and 60% (*n* = 85) were likely to use an intervention for child sleep support (Table 6). Sixty-one percent and 63% of parents reported being moderately/very/extremely likely to use a self-guided program or a therapist assisted program, respectively.

**Table 6.** Parent Interest in Online or Smartphone Interventions During COVID-19^a^.

	Moderately/very/extremely likely
	*N* (%)
How likely would you be to use an online or smartphone intervention for the following reasons:	
Mental health support for my child	100 (69.4)
Parenting support	100 (69.9)
Education support for my child	100 (69.4)
Sleep support for my child	85 (59.9)
For any of the above areas of assistance, how likely are you to use a:	
Self-guided internet or smartphone-app based program	86 (61.0)
Therapist-assisted internet or smartphone-app based treatment program	90 (62.9)

*Note*. ^a^*N* = 141–144.

## Discussion

Parents reported that the COVID-19 pandemic had contributed to worse functioning for their children across some aspects of physical health, media use, and mental health, however, some areas were unchanged (e.g., sleep, distractibility). The current cohort did not include any children or household members who had contracted COVID-19, and only three had a non-household family member diagnosed. Despite this, about one third of parents reported their child was substantially worried about themselves and others becoming infected. Most parents also reported positives associated with the COVID-19 pandemic. Child stress related to the COVID-19 restrictions was associated with negative changes in functioning across most mental health domains assessed such as general worry, enjoyment of activities, sad/depressed mood, fidgety behavior, distractibility, fatigue, irritability, loneliness, and negative thoughts. Additionally, COVID-19 stress related to restrictions was associated with increased social media use and there was some evidence of associations with reduced physical activity and outdoor time. In contrast, there was little evidence that worry about contracting COVID-19 was associated with negative changes in functioning. The adverse impacts experienced for children with ADHD in countries with higher infection rates may be even more significant.

We compared parent-reported child functioning prior to and during the COVID-19 pandemic with results somewhat consistent with the investigations in Shanghai (Zhang et al., 2020) and France (Bobo et al., 2020). Parents reported that during the pandemic children exercised less, had less outdoor time and had more screen time than 3 months prior to the pandemic. Children were reported to have increased sadness, less enjoyment in activities and increased loneliness during the pandemic. Taken together, these data suggest that there has been a change in experience for families of children with ADHD associated with the onset and first wave of COVID-19 in Australia.

Furthermore, we found that child COVID-19 stress was related to negative changes across most areas of functioning, particularly mental health functioning, even when accounting for pre-existing internalizing and/or externalizing disorder diagnoses and ADHD medication use. In contrast, there was little evidence that COVID-19 worries, such as fear of contracting the illness, was associated with poorer functioning. These findings suggest that the stress associated with lockdown (e.g., less social contact, cancellation of events and extra-curricular activities, changed routine) may be associated with poorer mental health in children with ADHD. Although this is the first study to our knowledge that has examined the association between child COVID-19 stress and functioning in children with ADHD, this finding is consistent with studies linking COVID-19-related risk factors (e.g., time spent thinking about COVID-19, perceived negative impact of COVID-19) to higher levels of mental health difficulties in children (Xie et al., 2020), adolescents (Ellis et al., 2020), and adults in the general population (Guo et al., 2020; Huang & Zhao, 2020; Li et al., 2020; Liu et al., 2020; Ustun, 2020).

We will track these changes over time to ascertain whether the pandemic is associated with enduring changes in these areas. It is anticipated that with the lifting of restrictions some of these changes may improve, however, strategies may be needed to assist in some areas; for example, developing a family media plan for reducing screen time (Dalope & Woods, 2018; Guram & Heinz, 2018) and using a “fading” approach to slowly reduce problematic media time and sedentary behavior to pre-pandemic levels. Such strategies could be delivered by health professionals via telehealth or even app-supported public health interventions. Of note, a relatively high proportion of families were interested in online or web-based support. Furthermore, given the links observed in this study between child COVID-19 stress and negative changes in functioning, helping children to cope with COVID-19 and associated lockdowns and restrictions is imperative. Resources developed specifically for children with neurodevelopmental disorders including ADHD most likely will be helpful.

We recommend that families are asked about stressors during clinical consultations and that brief supportive advice is provided or referrals for further assistance if needed. Nearly half of parents reported changes to their work situation, with the most common change being working from home. Over 30% of parents reported their child was experiencing moderate to extreme stress or concern related to COVID-19-related life challenges, such as restrictions on leaving home and changes in family and social contacts. Although the pandemic has been associated with substantial stress for some families, two-thirds of parents of children with ADHD reported some positive impacts. The positive aspects should be elicited when speaking to families and may be used as a source of strength and resilience (Dvorsky et al., 2020).

There were several areas where functioning was unchanged, on average. There were no differences in the proportion of children having less than 8 hours of sleep per day. Overall, about 40% of children with ADHD were receiving less than 8 hours of sleep per day irrespective of the COVID-19 pandemic, thus sleep problems already appeared to be elevated in this cohort. Moreover, no overall differences were found in the proportion of children feeling moderately/very anxious/nervous or worried. However, again overall rates of anxiety were high, with around 45% to 50% experiencing at least moderate symptoms both before and during the COVID-19 restrictions. This is consistent with the growing body of research reporting a high prevalence of sleep problems and anxiety in children with ADHD (Becker, 2020; Sciberras et al., 2014).

Alterations in the provision or quality of medical care during this phase of the pandemic were highlighted by families. A small proportion of families reported difficulty accessing medication (about 1 in 9) or health care services (about 1 in 8). Most children did not have a medication change during the period surveyed, in line with recommendations (Cortese et al., 2020). There were mixed results regarding parents’ appraisal of telehealth, with similar proportions of families rating telehealth as being the same (42%) or worse (48%) quality compared to face-to-face appointments. Given that there could be a shift to increased use of telehealth post-pandemic, the positives (e.g., increased accessibility), and negatives (e.g., reduced satisfaction), as well as individual differences in preference, need to be considered. Further research is needed to establish the efficacy of online approaches for child mental health difficulties (Fegert et al., 2020).

This study has several strengths. We examined a range of issues likely to be affecting children with ADHD and their families. Our sample is relatively large and comprises a diverse mix of children and adolescents with ADHD, with varying comorbidity profiles. There are also several limitations that should be considered. The first is that we rely on retrospective parent reports of pre-pandemic functioning. The absence of a control group means that we are unable to comment on how specific these effects are to children with ADHD versus children in the general population, as well as those with other neurodevelopmental and/or mental health disorders. Given the online nature of this study and resources available, confirmation of ADHD diagnosis and comorbid conditions using standardized diagnostic tools was not possible. Furthermore, we largely focused on child functioning and did not have data available on parents’ own COVID-19 stress and worries. It may be that parent ratings of their children’s functioning are influenced by parent functioning. Future research should aim to survey children specifically about their own functioning during the COVID-19 pandemic. Our recruitment through ADHD organizations and support groups may have resulted in a sample with relatively high interest in help seeking. Many characteristics of our sample were similar to a large clinical audit (*N* = 1528) of children with ADHD in Australia attending pediatric services, including the proportion of boys, language spoken at home, child age, most comorbidities (e.g., ODD, learning disorder), and medication use (Efron et al., 2013). However, in contrast to that audit, a larger proportion of children in this study were reported to have an anxiety disorder diagnosis. It is important to recognize that our survey will have only reached those who have access to the internet.

Further research is needed to track the pandemic-related functioning of children with ADHD over time, particularly as children transition back to school and as restrictions change. As of July 2020, one Australian state (Victoria) is experiencing a second rise in COVID-19 cases and an associated re-enforcement of strict social distancing restrictions; other states have maintained relatively low COVID-19 incidence and a reduced level of restrictions. We will follow this cohort of children longitudinally to understand the longer-term impacts of the COVID-19 pandemic and associated economic downturn on the functioning of children with ADHD and their families.

## Supplemental Material

sj-pdf-1-jad-10.1177_1087054720978549 – Supplemental material for Physical Health, Media Use, and Mental Health in Children and Adolescents With ADHD During the COVID-19 Pandemic in Australia Click here for additional data file. Supplemental material, sj-pdf-1-jad-10.1177_1087054720978549 for Physical Health, Media Use, and Mental Health in Children and Adolescents With ADHD During the COVID-19 Pandemic in Australia by Emma Sciberras, Pooja Patel, Mark A. Stokes, David Coghill, Christel M. Middeldorp, Mark A. Bellgrove, Stephen P. Becker, Daryl Efron, Argyris Stringaris, Stephen V. Faraone, Susannah T. Bellows, Jon Quach, Tobias Banaschewski, Jane McGillivray, Delyse Hutchinson, Tim J. Silk, Glenn Melvin, Amanda G. Wood, Anna Jackson, George Loram, Lidia Engel, Alicia Montgomery and Elizabeth Westrupp in Journal of Attention Disorders
