# Supplementary material for: Physical Health, Media Use, and Mental Health in Children and Adolescents With ADHD During the COVID-19 Pandemic in Australia
Source: J Atten Disord. 2020 Dec 17;26(4):549–62. doi: 10.1177/1087054720978549 (PMC8785303; doi:10.1177/1087054720978549)
Supplement: sj-pdf-1-jad-10.1177_1087054720978549 – Supplemental material for Physical Health, Media Use, and Mental Health in Children and Adolescents With ADHD During the COVID-19 Pandemic in Australia [file sj-pdf-1-jad-10.1177_1087054720978549.pdf]

Supplementary Tables

Table 1. *Expanded data on differences in current child physical health, media use and mental health compared to 3 months prior to COVID-19*

Table 2. *Association between COVID-19 stress and dimensional changes (current functioning compared to 3 months prior to pandemic) in physical health, media use and mental health*

Figure 1. *Confirmatory factor analysis - COVID-19 worry and COVID-19 stress variables*

Supplementary Table 1.

Expanded data on differences in current child physical health, media use and mental health compared to 3 months prior to COVID-19

	**Pre-COVID-19 *N***	**Pre-COVID-19 %[a]**	**During COVID-19[b] *N***	**During COVID-19[b] %[b]**
**Physical health**				
How many hours per night did your child sleep on average?				
Less than 6 hours	12	6.12	18	10.06
6-8 hours	63	32.14	52	29.05
8-10 hours	106	54.08	96	53.63
More than 10 hours	15	7.65	13	7.26
How many days per week did your child exercise for at least 30 minutes?				
None	12	6.12	23	12.85
1-2 days	36	18.37	71	39.66
3-4 days	51	26.02	36	20.11
5-6 days	37	19.39	20	11.17
Daily	59	30.10	29	16.20
How many days per week did your child spend time outdoors?				
None	2	1.02	9	5.03
1-2 days	21	10.71	59	32.96
3-4 days	32	16.33	24	13.41
5-6 days	43	21.94	28	15.64
Daily	98	50.00	59	32.96
**Media use**				
Time per day watching TV or digital media?				
No TV or digital media	0	0	1	0.56
Under 1 hour	36	18.85	15	8.47
1-3 hours	127	66.49	93	52.54

4-6 hours	18	9.42	54	30.51
More than 6 hours	10	5.24	14	7.91
Time per day using social media?				
No social media	124	65.26	99	55.93
Under 1 hour	30	15.79	25	14.12
1-3 hours	28	14.74	35	19.77
4-6 hours	6	3.16	11	6.21
More than 6 hours	2	1.05	7	3.95
Time per day playing video games				
No video games	54	28.27	40	22.60
Under 1 hour	59	30.89	32	18.08
1-3 hours	57	29.84	64	36.16
4-6 hours	12	6.28	27	15.25
More than 6 hours	9	4.71	14	7.91
**Mental health**				
How worried was your child generally?				
Not worried at all	25	13.23	22	12.64
Slightly worried	78	41.27	65	37.36
Moderately worried	58	30.69	54	31.03
Very worried	24	12.70	23	13.22
Extremely worried	4	2.12	10	5.75
How happy versus sad was your child?				
Very sad/depressed/unhappy	4	2.12	9	5.17
Moderately sad/depressed/unhappy	48	25.40	63	36.21
Neutral	55	29.10	48	27.59
Moderately happy/cheerful	69	36.51	40	22.99
Very happy/cheerful	13	6.88	14	8.05
How much had your child been able to enjoy his/her usual activities?				
Not at all	12	6.35	31	17.92
Slightly	26	13.76	76	43.93
Moderately	46	24.34	44	25.43
Very much	72	38.10	20	11.56
A lot	33	17.46	2	1.16
How relaxed versus anxious was your child?				

Very relaxed/calm	3	1.60	5	2.89
Moderately relaxed/calm	36	19.15	31	17.92
Neutral	46	24.47	42	24.28
Moderately nervous/anxious	85	45.21	73	42.20
Very nervous/anxious	18	9.57	22	12.72
How fidgety or restless was your child?				
Not fidgety/restless at all	6	3.17	7	4.05
Slightly fidgety/restless	39	20.63	30	17.34
Moderately fidgety/restless	74	39.15	51	29.48
Very fidgety/restless	54	28.57	45	26.01
Extremely fidgety/restless	16	8.47	40	23.12
How fatigued or tired was your child?				
Not fatigued or tired at all	31	16.58	24	13.79
Slightly fatigued or tired	63	33.69	47	27.01
Moderately fatigued or tired	68	36.36	55	31.61
Very fatigued or tired	20	10.70	35	20.11
Extremely fatigued or tired	5	2.67	13	7.47
How well was your child able to concentrate or focus?				
Very focused/attentive	4	2.13	8	4.60
Moderately focused/attentive	46	24.47	25	14.37
Neutral	24	12.77	26	14.94
Moderately unfocused/distracted	86	45.74	67	38.51
Very unfocused/distracted	28	14.89	48	27.59
How irritable or easily angered was your child?				
Not irritable or easily angered at all	9	4.79	9	5.17
Slightly irritable or easily angered	65	34.57	45	25.86
Moderately irritable or easily angered	55	29.26	44	25.29
Very irritable or easily angered	41	21.81	35	20.11
Extremely irritable or easily angered	18	9.57	41	23.56
How lonely was your child?				
Not lonely at all	71	37.77	32	18.39
Slightly lonely	66	35.11	45	25.86
Moderately lonely	34	18.09	54	31.03
Very lonely	10	5.32	32	18.39

Extremely lonely	7	3.72	11	6.32
To what extent did your child express negative thoughts or things that made them feel bad?				
Not at all	15	7.98	21	12.07
Rarely	51	27.13	41	23.56
Occasionally	78	41.49	64	36.78
Often	29	15.43	29	16.67
A lot of the time	15	7.98	19	10.92

[a]*N* ranges 187-196; [b]*N* ranges 173-179

Supplementary Table 2.

Association between COVID-19 stress and dimensional changes (current functioning compared to 3 months prior to pandemic) in physical health, media use and mental health

	**Adjusted COVID-19 Worries[a,b]**			**Adjusted COVID-19 Stress[a,c]**	
	**Coef (95% CI)**	**p**		**Coef (95% CI)**	**p**
**Physical health**					
Sleep duration	-0.13 (-0.24, -0.02)	.02		-0.06 (-0.17, 0.06)	.34
Physical activity	.05 (-0.17, 0.26)	.66		-0.40 (-0.63, -0.17)	.001
Outdoor time	.06 (-0.17, -.28)	.62		-0.31 (-0.55, -0.07)	.01
**Media use**					
Television	-0.03 (-0.17, 0.11)	.66		0.09 (-0.06, 0.23)	.25
Social media	-0.08 (-0.18, 0.03)	.14		0.19 (0.09, 0.30)	<.001
Gaming	0.11 (-0.03, 0.25)	.13		0.03 (-0.11, 0.18)	0.64
**Mental health**					
General worry	0.10 (-0.08, 0.29)	.27		0.45 (0.26, 0.64)	<.001
Sad/depressed	0.14 (-0.04, 0.33)	.13		-0.59 (-0.79, -0.40)	<.001
Enjoying activities	0.06 (-0.16, 0.28)	.59		-0.68 (-0.91, -0.45)	<.001
Anxious/nervous	-0.07 (-0.28, 0.13)	.47		0.57 (0.36, 0.78)	<.001
Fidgety	-0.07 (-0.24, 0.09)	.37		0.48 (0.32, 0.65)	<.001
Fatigue	-0.13 (-0.30, 0.05)	.16		0.40 (0.22, 0.59)	<.001
Distractibility	-0.08 (-0.29, 0.14)	.48		0.51 (0.28, 0.73)	<.001
Irritable	-0.16 (-0.37, 0.05)	.14		0.70 (0.48, 0.93)	<.001
Lonely	-0.07 (-0.26, 0.12)	.46		0.77 (0.57, 0.96)	<.001
Negative thoughts	-0.16 (0.34, .01)	.06		0.64 (0.46, 0.83)	<.001

[a] N=165-168; [b] Adjusted for COVID-19 stress, child age, child sex, ADHD medication use, externalizing disorder, internalizing disorder, neighbourhood socio-economic status, and financial insecurity; [c] Adjusted for COVID-19 worries, child age, child sex, ADHD medication use, externalizing disorder, internalizing disorder, neighbourhood socio-economic status, and financial insecurity.

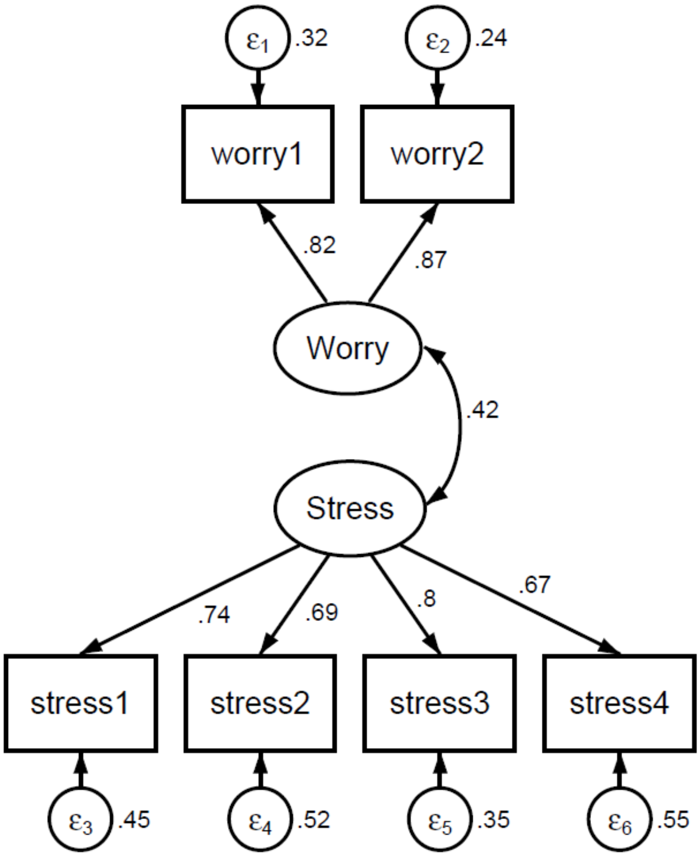

Figure 1. *Confirmatory factor analysis - COVID-19 worry and COVID-19 stress variables (n=200)*

Note: worry1=child worry about becoming infected, worry2=child worry about friends or family being infected, stress1=how stressful the restrictions on leaving home have been for the child, stress2= how stressful changes in family contacts have been for the child, stress3=how stressful changes in friend contacts have been for the child; stress4=difficulty experienced with cancellation of events.
